# Menstrual Effluent in the Pathogenesis and Diagnosis of Endometriosis—A Systematic Review

**DOI:** 10.3390/diagnostics16050677

**Published:** 2026-02-26

**Authors:** Rafał Watrowski, Stoyan Kostov, Eva Tsoneva, Sebastian D. Schäfer, Radmila Sparić, Mario Palumbo, Veronika Günther, Slavica Akšam, Angel Yordanov, Pierluigi Chieppa, Ingolf Juhasz-Böss, Salvatore Giovanni Vitale, Ibrahim Alkatout

**Affiliations:** 1 Department of Gynecology, Helios Hospital Müllheim, Heliosweg 1, 79379 Müllheim, Germany; 2 Faculty of Medicine, University of Freiburg, 79106 Freiburg, Germany; 3 Department of Gynecology, Hospital “Saint Anna”, 9002 Varna, Bulgaria; drstoqn.kostov@gmail.com; 4 Research Institute, Medical University Pleven, 5800 Pleven, Bulgaria; dretsoneva@gmail.com; 5 Department of Reproductive Medicine, Specialized Hospital for Active Treatment of Obstetrics and Gynaecology Dr Shterev, 1330 Sofia, Bulgaria; 6 Department of Gynecology and Obstetrics, Clemenshospital Münster, 48153 Münster, Germany; seb.schaefer@alexianer.de; 7 Faculty of Medicine, University of Belgrade, Dr Subotića 8, 11000 Belgrade, Serbia; radmila@rcub.bg.ac.rs (R.S.); slavicaaksam2012@gmail.com (S.A.); 8 Clinic for Gynecology and Obstetrics, University Clinical Centre of Serbia, Dr Koste Todorovića 26, 11000 Belgrade, Serbia; 9 Department of Public Health, School of Medicine, University of Naples Federico II, 80138 Naples, Italy; mpalumbo@gmail.com; 10 Department of Obstetrics and Gynecology, University Hospitals Schleswig-Holstein, Campus Kiel, 24105 Kiel, Germany; veronika.guenther@uksh.de (V.G.); ibrahim.alkatout@uksh.de (I.A.); 11 Department of Gynecologic Oncology, Medical University Pleven, 5800 Pleven, Bulgaria; angel.jordanov@gmail.com; 12 Department of Surgical Sciences, Gynecology and Obstetrics 1, A.O.U. City of Health and Science of Turin, S. Anna Hospital, 10126 Turin, Italy; pchieppa@libero.it; 13 Department of Obstetrics and Gynecology, Medical Center—University Hospital Freiburg, 79106 Freiburg, Germany; ingolf.juhasz-boess@uniklinik-freiburg.de; 14 Obstetrics and Gynecology Unit, Department of General Surgery and Medical Surgical Specialties, “Gaspare Rodolico” University Hospital, University of Catania, 95124 Catania, Italy; vitalesalvatore@hotmail.com

**Keywords:** endometriosis, endometrium, menstrual effluent, menstrual blood, biospecimen, biomarkers, non-invasive diagnosis, aromatase, VEGF, progesterone resistance

## Abstract

**Background:** The individual and social burden of endometriosis is high, and the diagnosis is usually delayed by 7–10 years. Menstrual effluent (ME) represents an accessible and uniquely informative biofluid. This systematic review evaluated the pathophysiological relevance and diagnostic potential of ME in endometriosis. **Methods**: Following PRISMA 2020 guidelines, we systematically searched PubMed/MEDLINE, EBSCOhost (Academic Search Premier, APA PsycArticles, APA PsycInfo, CINAHL, and MEDLINE), Semantic Scholar, and Google Scholar from inception to 30 November 2025. Original studies analyzing human ME or ME-derived cells in women with endometriosis versus controls were eligible. We extracted study design, analytic methods, diagnostic accuracy metrics (AUC, sensitivity, and specificity), mechanistic pathways, and risk of bias (QUADAS-2 for diagnostic, and NIH tools for mechanistic studies). **Results:** Thirty-five studies were included. ME consistently captured key pathophysiological mechanisms of endometriosis, including impaired decidualization and progesterone resistance, immune dysregulation with diminished cytotoxic clearance, pro-angiogenic and invasive phenotypes, heightened stem/progenitor cell survival, cellular senescence and DNA damage, and altered extracellular-vesicle signaling. Diagnostic accuracy was reported in nine studies. Aromatase mRNA showed the highest performance (AUC 0.977), followed by TGF-β1 (AUC 0.973) and IGFBP1 (AUC 0.92). A lipidomic two-marker model achieved an AUC of 0.87. All diagnostic assessments were based on case–control studies; none conducted prospective validation. **Conclusions:** ME is a biologically relevant, non-invasive, and patient-acceptable biospecimen reflecting core endometriosis mechanisms and yielding promising diagnostic accuracy. The highest diagnostic performance was achieved for assays reflecting steroidogenic and growth-factor pathways (e.g., aromatase and TGF-β1). Standardization and prospective validation are needed before clinical adoption.

## 1. Introduction

Endometriosis is a chronic, estrogen-dependent inflammatory disease defined by the presence of endometrium-like tissue outside the uterine cavity [1]. It affects approximately 10% of women of reproductive age [1]. Despite global incidence variation due to biological and diagnostic factors, a rising burden has been observed over the past three decades [2].

Although retrograde menstruation remains the most established etiological model, current concepts increasingly recognize that the eutopic endometrium itself may be altered even before lesion formation (“endometrium as the first culprit of endometriosis”). These changes include deregulated gene expression, enhanced proliferative, adhesive, and angiogenic potential, aberrant cytokine release, impaired apoptotic regulation, and altered immune interactions [3,4,5].

Consequently, the pathogenesis of endometriosis can be viewed as a cascade where the nature of the transported tissue is as critical as the transport mechanism itself [6]. This sequence is likely initiated by uterine hyperperistalsis and tissue injury and repair (TIAR) mechanisms, which generate a “super-charged” menstrual effluent (ME) characterized by progesterone resistance, stem cell abundance, and invasive potential (the *altered seed*) [7]. Following retrograde menstruation (the *transport*), specific factors carried within this effluent—such as TGF-β1, VEGF, and immune-suppressive cytokines—actively condition the peritoneal microenvironment (the *soil*) [8,9]. By inducing macrophage reprogramming and mesothelial conditioning, the ME creates its own hospitable niche, allowing the “altered” tissue to evade immune clearance and establish ectopic lesions [6,10,11,12].

Recent evidence further characterizes endometriosis as a systemic disease with multiple comorbidities, involving neuroendocrine, immunological, metabolic, and inflammatory disturbances that extend beyond the local pelvic environment [13,14]. The disease presents with a broad spectrum of symptoms including nociceptive, neuropathic, and nociplastic pelvic pain, dysmenorrhea, dyspareunia, subfertility, gastrointestinal symptoms (“endobelly”), chronic fatigue, and psychological comorbidity such as anxiety and depression [15,16,17]. These manifestations significantly impair health-related quality of life and are often under-addressed in routine clinical care [18,19,20]. The coexistence of endometriosis with adenomyosis is up to 80%, and the pathogenetic mechanisms and clinical presentations are shared [21,22,23]. The socioeconomic burden is substantial, with high healthcare utilization, work absenteeism, and annual costs estimated at >10,000 EUR per patient [24].

Laparoscopy remains the reference standard, particularly in “see-and-treat” clinical contexts [1,25]. However, current diagnostic strategies increasingly prioritize non-invasive assessment, favoring transvaginal sonography (TVS) and magnetic resonance imaging (MRI) [26,27,28,29]. Despite this recommendation shift, imaging-based diagnosis is primarily limited to detecting deep infiltrating endometriosis, whereas superficial, lateral pelvic or extrapelvic lesions remain largely undetectable. Diagnostic accuracy is dependent on lesion location and examiner expertise [30,31,32], with considerable interobserver variability [33]. In one study, TVS demonstrated a sensitivity of only 61% (95% CI 49–72%), and a specificity of 94% (95% CI 71–100%), but a negative predictive value as low as 36% [34].

A Cochrane review concluded that no current imaging modality fulfills the criteria for a replacement or triage test compared with laparoscopy [25]. Thus, although current guidelines increasingly promote non-invasive diagnostic strategies, mainly via imaging-based approaches, these remain confined to structural visualization and fail to capture underlying biological disease activity. Given the substantial diagnostic delay and the invasive nature of surgical confirmation, there is an urgent clinical need for reliable, biologically based, non-invasive biomarkers. As in other diseases, biomarkers of endometriosis should be reliable and reproducible, but also acceptable for patients, easily obtainable, and cost-effective [35,36]. Unfortunately, only few circulating biomarkers, including serum CA-125, inflammatory proteins (S100-A12), and proteomic patterns, have demonstrated moderate diagnostic utility but with limited reproducibility and specificity [37,38]. In peritoneal fluid, nitric oxide [39], phenylalanyl-isoleucine [40], fibronectin, or collagen IV [41] are significantly elevated in endometriosis, but peritoneal-fluid sampling is inherently invasive or, if performed during laparoscopy, of questionable utility. Alternative biofluids such as urine, saliva, and ME have therefore gained attention [29,38,42]. Molecular approaches—such as the salivary miRNA signature validated by Bendifallah et al. [43], with a sensitivity of 97.3% and a specificity of 94.1%—provide a proof of concept that biologically informed diagnostics may outperform purely imaging-based assessment.

In this context, ME represents a uniquely informative biospecimen reflecting cellular and molecular features at the moment of retrograde dissemination [44]. Unlike peripheral blood, ME contains intact stromal and local immune cells, epithelial elements, extracellular vesicles, and tissue fragments mirroring intrauterine biology [45]. Importantly, ME may capture pre-lesional aberrations at the moment they enter the pelvic cavity via retrograde menstruation, potentially reflecting early pathogenic characteristics including inflammatory activation, immune evasion, cellular resilience, or defective decidualization. This aligns with the emerging hypothesis that endometrial dysfunction precedes lesion formation and therefore may be detectable using ME-based testing [44,45,46].

Therefore, systematic evaluation of ME may enable simultaneous insight into both disease mechanisms and diagnostic performance, providing a biologically grounded approach that complements and potentially enhances current imaging-based strategies.

The aim of this systematic review was to examine the available evidence on the role of ME in diagnosing and understanding endometriosis, with specific focus on (i) evaluating diagnostic biomarker performance, (ii) identifying mechanistic alterations reflected in ME, and (iii) assessing methodological considerations relevant for future translation into clinical practice.

## 2. Materials and Methods

### 2.1. Protocol and Registration

This systematic review was conducted in accordance with the Preferred Reporting Items for Systematic Reviews and Meta-Analyses (PRISMA) 2020 guidelines (Supplementary Materials) [47]. All methodological steps—including search strategy, eligibility criteria, screening procedure, data extraction, and synthesis framework—were defined a priori and consistently applied throughout the review.

### 2.2. Information Sources and Search Strategy

A systematic literature search was conducted in PubMed/MEDLINE, Semantic Scholar, and via the EBSCOhost platform (including Academic Search Premier, APA PsycArticles, APA PsycInfo, CINAHL, and MEDLINE) from database inception to 30 November 2025. The PubMed search combined Medical Subject Headings (MeSH) with free-text terms and was structured as follows: *(“Endometriosis”[MeSH] OR “Endometriosis”) AND (“menstrual effluent” OR “menstrual blood” OR “menstrual discharge” OR “menstrual endometrium” OR “dried menstrual spots”).*

Semantic Scholar was searched using the search string *“Endometriosis” AND (“menstrual effluent” OR “menstrual blood” OR “menstrual discharge” OR “menstrual endometrium” OR “dried menstrual spots”)*, whereas EBSCOhost was searched using the combination of terms: *“Endometriosis” AND (“menstrual effluent” OR “menstrual blood”)*.

This query retrieved:

• 230 records in PubMed
• 347 records via EBSCOhost
• 167 records in Semantic Scholar

No filters were applied regarding language, publication year, study size, or methodology. In addition, Google Scholar was searched on 30 November 2025 using the query (“Endometriosis” AND “menstrual effluent”), sorted by relevance; all retrieved records (*n* = 963) were screened by title. Search results from PubMed/MEDLINE, EBSCOhost, and Semantic Scholar were exported to Zotero (Corporation for Digital Scholarship, Vienna, VA, USA), and duplicates were removed prior to screening.

### 2.3. Study Selection

A total of 744 records were identified from databases. After removal of duplicates (*n* = 506), 238 unique records underwent title and abstract screening by two independent reviewers (R.W., S.K.). After exclusion of clearly irrelevant studies (*n* = 191), 47 full-text articles were assessed for eligibility.

Of these:

• 35 studies met all inclusion criteria and were included in the final synthesis.
• 12 were excluded due to (i) insufficient use of ME or menstrual blood, (ii) absence of comparative data between endometriosis and control groups, (iii) use of unrelated cellular models, or (iv) insufficient reporting for reproducible data extraction.

**Figure 1:**
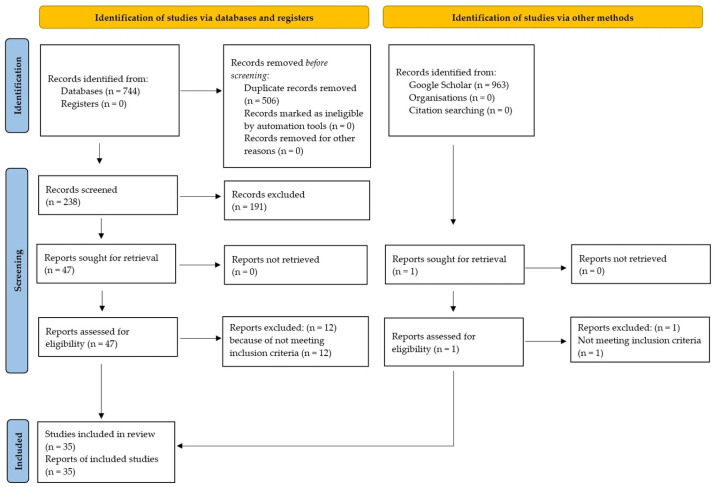
PRISMA 2020 flowchart of study identification and selection.

Disagreements during screening were resolved through discussion with third evaluators (R.S., E.T., and S.G.V.). The selection process has been summarized in the PRISMA 2020 flow diagram (Figure 1).

### 2.4. Eligibility Criteria

Studies were eligible if they: (a) used human ME or ME-derived cellular fractions (e.g., stromal cells, immune cells, extracellular vesicles) as the primary biospecimen; (b) included human participants of reproductive age with endometriosis confirmed by laparoscopy and/or histopathology, and compared them to an appropriate control group (asymptomatic, laparoscopically confirmed disease-free, or self-reported healthy controls); (c) reported original data on molecular, cellular, functional, or multi-omics analyses relevant to endometriosis pathophysiology or diagnosis; (d) presented comparative results between endometriosis and control groups based on the human ME-derived material; and (e) provided sufficient methodological detail to allow reproducible data extraction (including the analytical method and reporting units for quantified biomarkers). Eligible study types included diagnostic accuracy investigations, reporting area-under-the-curve (AUC), sensitivity, specificity or cut-off values, and mechanistic research focused on the role of ME in the pathogenesis of endometriosis (e.g., decidualization capacity, immune dysfunction, scRNA-seq-based profiling), but often holding potential diagnostic relevance.

Studies were excluded if they: (a) used endometrial biopsies, peritoneal fluid, or serum without concurrent analysis of ME; (b) relied exclusively on non-human biospecimens (e.g., rodent ME, primate models); (c) used non-human cell lines or xenografts without originating from human ME; (d) were case reports, reviews, conference abstracts, or methodological papers without a human endometriosis/control cohort; or (e) lacked sufficient reporting of biomarker quantification methods and/or measurement units to enable reproducible extraction and comparison. Studies combining human ME collection with secondary experimental models (e.g., in vitro functional assays, murine xenografts, or mechanistic validation in animal models) were retained, provided the primary biospecimen and comparative human data originated from ME of endometriosis patients and controls.

### 2.5. Data Extraction

Data were independently extracted by two reviewers (R.W. and S.K.) using a pre-defined template covering: (a) study design and population characteristics; (b) sample size (cases vs. controls), diagnosis confirmation method, and control type; (c) timing and method of ME collection and processing; (d) analytical methods (e.g., qPCR, scRNA-seq, ELISA, proteomics, functional assays); (e) diagnostic performance metrics (AUC, sensitivity, specificity, and thresholds where provided); (f) mechanistic insights (e.g., hormone signaling, decidualization defects, immune alteration, ECM remodeling); and (g) reported clinical implications and authors’ recommendations. Any discrepancies were resolved through discussion and cross-checking against the full texts.

### 2.6. Risk of Bias and Methodological Quality Assessment

Risk of bias was assessed separately for diagnostic-accuracy studies and for mechanistic or exploratory observational studies. For diagnostic-accuracy studies (i.e., studies reporting sensitivity, specificity, AUC and/or predefined cut-offs for ME-based markers), we used the domains of the QUADAS-2 framework: (a) patient selection, (b) index test, (c) reference standard, and (d) flow and timing. Each domain was judged as low, high, or unclear risk of bias. Given the predominance of case–control designs and surgically selected populations, special attention was paid to spectrum bias and applicability. For mechanistic and non-diagnostic observational studies, we used a simplified, NIH-inspired approach based on the NIH Quality Assessment Tools for observational cohort/cross-sectional and case–control studies. We evaluated: (a) study population and selection, (b) exposure/biomarker measurement, (c) outcome ascertainment, (d) control of confounding (where applicable), and (e) reporting/analysis clarity. These domains were also graded as low, moderate, high, or unclear risk of bias.

### 2.7. Data Synthesis

Given the diversity of analytical techniques and outcome measures, meta-analysis was not appropriate. Instead, we applied structured qualitative synthesis, grouping findings according to predefined categories:

• Diagnostic markers were synthesized narratively and tabulated including reported sensitivity, specificity, AUC, and cut-offs where available. AUC 95% confidence intervals were extracted from the original publications where reported; otherwise, they were approximated from the AUC and case/control sample sizes using the Hanley–McNeil method, implemented in R via the JASP R-syntax module (JASP statistical software version 0.95.4; JASP Team, 2025)
• Mechanistic studies were grouped according to biological domains: stromal decidualization/progesterone resistance, immune dysregulation, extracellular remodeling and angiogenesis, metabolic elements (lipidomics), and multi-omic integration.
• When findings overlapped across platform types (e.g., reduced IGFBP1^+^ stromal cells seen both in ME culture and scRNA-seq), this was highlighted to enhance trans-method validation.

Tables were constructed to summarize general study characteristics (Table 1), diagnostic accuracy (Table 2), and mechanistic insights (Table 3). Narrative synthesis aligned with the review objectives.

## 3. Results

### 3.1. Study Characteristics

A total of 35 studies met the inclusion criteria (Table 1). Publication years ranged from 1990 to 2025, with a clear increase in studies published after 2020 [48,49,50,51,52,53,54,55,56,57,58,59,60,61,62,63,64,65,66,67,68,69,70,71,72,73,74,75,76,77,78,79,80,81,82]. Study designs were predominantly case–control, with all included studies analyzing ME or ME-derived cells. Sample sizes varied widely, from small exploratory cohorts (e.g., 7 vs. 7 in [74]) to moderate-sized case–control studies with a total of 40–70 participants (e.g., [55,56,57,75]) or more [71,73].

Endometriosis was confirmed surgically in 34 studies, while one study relied on symptom profile [69].

Control groups consisted mainly of healthy individuals without pelvic pain [55,56,58,60,61,68,69,70,75], with several studies including symptomatic but endometriosis-negative controls [52,59,81].

ME was predominantly collected using menstrual cups, typically during the first 24–48 h of menstruation, although a minority of studies used pads [55,58], a Cusco speculum [56], aspiration by syringe [61,67,68,77,81,82] or a pipelle [62,65,79]. As shown in Table 1, analytical approaches varied widely and included ELISA-based protein quantification, RT-qPCR-based assays, immunocytochemistry, lipidomics, single-cell RNA sequencing, proteomics, and functional decidualization assays.

No study included a prospective validation cohort, and all diagnostic analyses were conducted in case–control designs [55,56,60,68,69,70,75,81,82]. Mechanistic investigations focused on stromal-cell function [48,50,65], immune signatures [53,64,66], progenitor cell profiles [67], senescence [48,50], angiogenesis-related factors [69,71,72], extracellular vesicles [52,62], or multi-omic regulation [61]. The risk-of-bias rating for each included study is presented in Table A1 (Appendix A). Overall, most diagnostic studies were at high risk of bias in patient selection (case–control designs, surgical populations) and unclear risk regarding the blinding of index-test interpretation. Mechanistic studies were predominantly moderate-risk, driven by small sample sizes and limited control of confounding, but with generally robust laboratory methods and clearly reported outcomes.

### 3.2. Diagnostic Findings

Diagnostic performance related to ME analysis was reported in nine studies [55,56,60,68,69,70,75,81,82], all of which provided numerical diagnostic metrics such as sensitivity, specificity, AUC, or defined cut-offs (Table 2). These studies predominantly evaluated molecular markers, protein concentrations, functional stromal-cell assays, or lipidomic signatures in case–control settings with laparoscopically confirmed endometriosis and healthy controls. Several additional studies investigated ME-based biomarkers in a diagnostic context but did not report ROC values, sensitivity/specificity, or diagnostic thresholds. These exploratory diagnostic studies are summarized in Appendix A (Table A2) [51,57,58,59,61,66,67,71,72,73,74,76,77,80]. Two early studies evaluated the diagnostic performance of CA-125 in ME. In women with chronic pelvic pain, ME-CA-125 at the threshold of ≥72,000 U/mL identified endometriosis with a sensitivity of 89.3% and a specificity of 96.3% [81]. At the threshold of 100,000 U/mL, ME-derived CA-125 differentiated women from those without endometriosis with a sensitivity of 65.7% and a specificity of 89.3%, showing elevated ME-CA-125 levels across all endometriosis stages (sensitivity ranging from 60% for Stages I/II to 72.2% for Stages III/IV) and substantially outperforming serum CA-125 in the same population (sensitivity 32.5%, specificity 80.3%) [82]. These findings established an early proof-of-concept for ME-based biomarkers.

Across the nine studies with formal diagnostic metrics, discriminatory performance was highest for molecular markers related to steroidogenesis and inflammatory growth factors, as well as for functional stromal-cell assays and one lipidomic model. Aromatase mRNA expression in ME achieved the strongest individual performance, with an AUC of 0.977, sensitivity of 95%, and specificity of 90% at a defined expression ratio >1.63 [55]. SF-1 (AUC 0.862) and HSD17B2 (AUC 0.807) also demonstrated clinically relevant discriminatory ability, supporting the concept that aberrant hormonal gene expression and local hyperestrogenism are detectable in ME [55].

Protein biomarkers quantified via ELISA showed variable but generally moderate-to-high diagnostic potential. TGF-β1 concentration reached an AUC of 0.973 (95% CI 0.928–1.000), with sensitivity of 80% and specificity of 90% at a predefined threshold of 515 ng/mL [60]. VEGF-A levels distinguished cases from controls with sensitivity and specificity of 84.2% and 85.7%, respectively, and an AUC of 0.853; however, the small control group (38/7) increases uncertainty, especially for specificity [69]. Another study, analyzing VEGF staining intensity in endometrial cells from ME using immunocytochemistry, reported an AUC of 0.672 with low sensitivity (40%) but high specificity (93.33%) at a histoscore cut-off > 6 [68].

Functional assays based on ME-derived stromal cells also performed well. A decidualization-based test quantifying IGFBP1 secretion reached an AUC of 0.92 (95% CI 0.82–1.00), differentiating endometriosis from controls with 87.5% sensitivity and 91.7% specificity [70]. PR-B mRNA expression analyzed by qRT-PCR demonstrated 90.5% sensitivity and 81.0% specificity at a defined optical-density threshold of ≤1.1355 μg/dL [75]. However, the original publication contains inconsistent labeling between PR-B receptor expression and progesterone hormone concentration, and the reported unit (μg/dL) is unconventional for qRT-PCR data; the diagnostic metrics should therefore be interpreted with caution [75].

In addition, a lipidomic two-lipid model based on cardiolipin CL 16:0_18:0_22:5_22:6 and plasmenylphosphatidylethanolamine PE P-16:0/18:1 in dried menstrual blood spots achieved an AUC of 0.87 in cross-validated ROC analysis, with 81% sensitivity and 85% specificity at an optimal threshold of 0.59 [56]. This model was derived from 23 women with histologically verified endometriosis and 16 controls and indicates that lipid signatures in ME can carry independent diagnostic information.

To summarize, high diagnostic accuracy was most consistently achieved in:

(a) Molecular markers of hormonal regulation
  • Aromatase (AUC 0.977) [55];
  • TGF-Β1 (Auc 0.973) [60];
  • SF-1 (Auc 0.862) [55];
  • HSD17B2 (Auc 0.807) [55];
  • Marker combinations: aromatase and SF-1 0.92 (AUC 0.92), aromatase and HSD17B2 (AUC 0.89), SF-1 and HSD17B2 (0.83), and AUC 0.88 for all three markers [55].
(b) Functional stromal-cell assays
  • Decidualization response via IGFBP1 secretion (AUC 0.92) [70];
  • PR-B expression (sens 90.5%, spec 81.0%) [75].
(c) Protein-based markers
  • CA-125 (sens 65.7%, spec 89.3% at >100,000 U/mL [82]; sens 89.3%, spec 96.3% at ≥72,000 U/mL [81]);
  • VEGF-A (sens 84.2%, spec 85.7%; Auc 0.853) [69].
(d) Lipidomic signatures
  • Two-lipid model (CL 16:0_18:0_22:5_22:6 + PE P-16:0/18:1) from dried menstrual blood spots (AUC 0.87, sens 81%, spec 85%, threshold 0.59) [56].

In contrast, a large group of mechanistic studies identified statistically significant biomarker differences (e.g., proteomic, transcriptomic, immune, and stromal-cell signatures) without providing ROC-derived diagnostic metrics. These studies (Appendix A (Table A2)) suggest diagnostic potential but cannot be quantitatively assessed (e.g., [61,64]).

No study performed prospective validation, head-to-head comparison with imaging modalities, or real-world diagnostic testing. All diagnostic data were generated in small case–control cohorts, and none evaluated multi-marker algorithms in an independent population.

### 3.3. Mechanistic Findings

Mechanistic alterations detectable in ME or ME-derived cells were examined across molecular, cellular, immunological, proteomic, transcriptomic, and lipidomic approaches (Table 3) [48,49,50,52,53,55,56,58,61,63,64,65,66,67]. Although the heterogeneity of the methods precluded quantitative synthesis, several reproducible mechanistic themes emerged.

#### 3.3.1. Impaired Decidualization and Progesterone Resistance

Multiple studies demonstrated reduced decidualization capacity of ME-derived stromal cells, accompanied by lower IGFBP1 secretion [63,70,74], altered expression of progesterone-responsive genes [63], and diminished PR-B levels [75]. These findings are consistent with impaired progesterone signaling, a hallmark of endometriosis pathophysiology. Functional decidualization defects were observed both in diagnostic studies (PR-B analysis, decidualization assays) and in mechanistic investigations evaluating cellular responses to hormonal or inflammatory stimuli [55,58,74].

#### 3.3.2. Immune Dysregulation and Inflammatory Signaling

Several studies reported altered immune signatures within ME, indicating dysregulated innate and adaptive immune responses in endometriosis. These alterations included increased neutrophil activation and aging phenotypes [53], expansion or transcriptional reprogramming of Th17-associated immune profiles [64], reduced frequencies of perforin-positive CD8^+^ cytotoxic T cells [66], and disrupted macrophage-related signaling pathways [64]. These immune alterations were not uniform across studies, reflecting methodological differences (flow cytometry vs. transcriptomics) and heterogeneous immune compartments analyzed. Cytokine and mediator profiling further demonstrated elevations in pro-inflammatory factors alongside relative reductions in anti-inflammatory or regulatory signals in endometriosis samples [77].

#### 3.3.3. Angiogenesis and Extracellular Matrix (ECM) Remodeling

Proteomic, cytokine-based, and immunocytochemical studies identified alterations in angiogenesis-related factors and ECM-remodeling enzymes within ME, involving VEGF, endoglin (CD105), matrix metalloproteinases (mostly MMP-9), and their inhibitors [68,69,71,72,76,77]. An imbalance between MMP-9 and TIMP-1 was repeatedly noted [71,76], suggesting enhanced matrix-degrading capacity, although effect sizes and diagnostic discriminability varied across cohorts. The expression of VEGF or VEGF-A was increased in endometriosis-associated menstrual samples [69,72]; however, modest or non-significant differences were also reported [77,80].

#### 3.3.4. Stem/Progenitor Cell Populations

Evidence for altered stem/progenitor cell populations in ME includes increased clonogenic endometrial cell subsets—encompassing mesenchymal stromal and epithelial progenitor fractions—in women with endometriosis [67], supporting the concept that ME contains an expanded pool of regeneration-competent cells with enhanced survival or implantation potential. Beyond abundance, the stromal/stem cell compartment in endometriosis is characterized by a lesion-supportive biological program, with concomitant signals consistent with inflammatory activation, matrix remodeling/invasiveness, pro-angiogenic drive, and relative apoptosis resistance [62,78]. These findings position ME-derived progenitor/stromal cells as plausible “seed” contributors linking uterine-origin dysfunction with downstream establishment and persistence after retrograde dissemination [67].

#### 3.3.5. Cellular Adhesion and Peritoneal Interaction

Menstrual endometrial stromal cells (ESCs) from women with endometriosis showed higher adherence to LP9 peritoneal mesothelial cells (PMCs) than controls (43% vs. 32%), while epithelial cells (EECs) showed a similar trend that did not reach statistical significance (23% vs. 15%) [79]. This pro-adhesive phenotype was accompanied by more frequent expression of CD44 variant isoforms involved in hyaluronan binding (CD44v6–v9), particularly in ESCs [79]. In cultured ME-derived stromal stem cells (MenSCs), endometriosis-derived cells displayed higher expression of CD9, CD10, and CD29 and showed increased proliferation and Matrigel invasion, although adhesion to fibronectin-coated plates was not significantly different [78]. These findings support the concept that ME in endometriosis exhibits a more invasive and adhesive phenotype, facilitating ectopic implantation after retrograde menstruation.

#### 3.3.6. Cellular Senescence and DNA Damage Accumulation

Two studies [48,50] identified markers of premature cellular senescence and compromised genomic stability in ME-derived stromal cells from women with endometriosis. Reported alterations included impaired DNA damage repair responses, accumulation of senescence-associated markers, and dysregulation of p53-dependent stress signaling.

#### 3.3.7. Cellular and Molecular Heterogeneity in ME

Single-cell transcriptomic analyses showed disease-associated immune-stromal interactions, expanded inflammatory and dysfunctional cell states, and altered cellular proportions within ME from affected individuals [49,59,63]. In parallel, proteomic and lipidomic investigations identified discriminative metabolic, inflammatory, and structural protein patterns, as well as altered lipid species, distinguishing endometriosis from control samples [56,61]. In addition, analyses of ME-derived extracellular vesicles demonstrated disease-related changes in protein cargo implicated in immune modulation and tissue repair, suggesting that vesicle-mediated intercellular signaling contributes to the pathophysiology captured in ME [52]. One proof-of-concept study revealed ME not only as a diagnostic specimen but also as a source for cell-free therapeutic interventions. Exosomes derived from healthy menstrual stromal cells (NE-MenSCs) reduced the expression of inflammatory (IL-6, IL-8, IL-1β, COX-2, NF-κB, and TNF-α), proliferative (cyclin D1), migratory (MMP-2 and MMP-9), and angiogenic (VEGF) markers in endometriosis-derived MenSCs, and induced apoptosis of E-MenSC [62].

## 4. Discussion

This systematic review provides the most comprehensive synthesis to date of 35 studies evaluating ME as a source of diagnostic and pathophysiologic (mechanistic) information in endometriosis. We found consistent evidence that ME acts as a “liquid biopsy” of the eutopic endometrium, reflecting central pathophysiological abnormalities such as progesterone resistance, immune dysregulation, and altered cellular kinetics. Importantly, these biological signals translate into promising diagnostic performance. Several molecular and functional assays demonstrated area-under-the-curve (AUC) values exceeding 0.90 [55,60,70], supporting the concept that ME is a biologically meaningful, non-invasive tissue source for discriminating affected individuals from controls.

Mechanistically, the most consistently reported abnormality was impaired decidualization associated with progesterone resistance. Reduced IGFBP1 secretion, diminished PR-B expression, and dysregulation of progesterone-responsive genes were identified in both mechanistic [54,55] and diagnostic investigations [70,74,75]. These findings indicate that stromal-cell dysfunction is intrinsic to the eutopic endometrium and remains detectable during menstruation. Immune dysregulation was another hallmark feature. Findings included pro-inflammatory shifts such as increased neutrophil activation, Th17 expansion, aberrant macrophage polarization, and reduced cytotoxic CD8^+^ T-cell frequency [49,53,64,66,77]. These alterations align with the hypothesis that impaired immune clearance and heightened inflammatory tone in shed tissue promote the persistence of refluxed endometrial fragments. Similarly, markers of angiogenic signaling (VEGF and endoglin) and extracellular matrix remodeling (MMP-9 and TIMP-1) were consistently altered [69,71,72], reflecting an invasive phenotype.

Further biological complexity was revealed by studies identifying stem/progenitor cell abnormalities [67,73], premature cellular senescence with genomic instability [48,50], and distinct extracellular vesicle cargo [52]. Multi-omic and single-cell analyses confirmed that these disease-associated signatures—including altered immune-stromal interactions—are robustly mapped in ME [49,59]. The enhanced adhesive and invasive properties of menstrual endometrial cells from affected women [78,79] provide cellular-level validation of the “altered seed”. Differential expression of CD44 splice variants and adhesion molecules (CD9, CD10, and CD29) equips these cells with superior capacity to attach to peritoneal mesothelium and invade underlying stroma [78,79]. The observation that exosomes from healthy MenSCs can partially reverse the pathological phenotype of endometriosis-derived cells [62] further supports the concept that these cellular differences are modifiable and may represent therapeutic targets. These intrinsic cellular differences, combined with progesterone resistance and immune dysregulation, create a multi-level pathophysiological cascade detectable in ME.

### 4.1. Diagnostic Performance

Assays targeting these core disease mechanisms achieved the highest discriminatory accuracy. Among the nine studies reporting formal test metrics, molecular markers of hormonally regulated pathways performed best. Aromatase mRNA (AUC 0.977), TGF-β1 protein (AUC 0.973), and SF-1 mRNA (AUC 0.862) showed excellent diagnostic potential [55,60]. Functional assays assessing stromal-cell decidualization (IGFBP1; AUC 0.92) and receptor status (PR-B; sensitivity 90.5%) also demonstrated high diagnostic accuracy [70,75]. However, the diagnostic performance reported for PR-B [75] should be interpreted cautiously, as the original publication contains reporting inconsistencies regarding the measured analyte and its unit.

Lipidomic profiling offered promising sensitivity (81%) and specificity (85%) based on a two-lipid model (cardiolipin CL 16:0_18:0_22:5_22:6 plus PE P-16:0/18:1), further supporting the biological plausibility of ME-based diagnostics [56]. Conversely, simple immunocytochemical staining showed high specificity but lower sensitivity, suggesting that functional and molecular assays provide superior diagnostic value compared to morphology alone [68,73]. Despite these promising results, the risk of bias was rated as high across all diagnostic studies due to case–control designs that recruited surgically confirmed patients and healthy or asymptomatic controls. No study included a prospective validation cohort or assessed performance in an unselected, real-world population. Given the high risk of spectrum bias inherent to case–control designs, reported AUC values should be interpreted as proof-of-concept rather than as direct estimates of clinical diagnostic performance in routine care settings.

Moreover, the potential of ME-based testing is not restricted to distinguishing endometriosis from health. A reliable non-invasive assay could also help identify endometriosis in oligosymptomatic or atypical presentations. A particular unmet need concerns patients presenting with gastrointestinal symptoms (often referred to as “endo belly” or misattributed to irritable bowel syndrome) [18,83] or with bladder pain syndrome/interstitial cystitis [84]. These presentations are often not initially considered gynecological, which can contribute to unnecessary investigations, higher costs, and diagnostic delay. A screening test that helps rule out or support endometriosis in patients with diffuse gastrointestinal symptoms or chronic bladder-associated pain could meaningfully improve diagnostic pathways and quality of life for many affected women.

### 4.2. Clinical Implications

The ability to non-invasively access cellular and molecular signatures of endometriosis offers a significant advantage over current diagnostic pathways. Definitive diagnosis often relies on laparoscopy, contributing to delays of several years. ME-based testing could shorten this interval, reduce the disease burden and save costs. Because markers like IGFBP1 and PR-B reflect intrinsic progesterone resistance—a process believed to precede overt lesion formation—they may offer utility for early detection and risk stratification even in the absence of visible lesions on imaging. Furthermore, inflammatory signatures (TGF-β1, cytokines) or lipidomic profiles might help distinguish endometriosis from other causes of pelvic pain [56,60]. These approaches are complementary to analyses of extracellular-vesicle cargo and non-coding RNA signatures as emerging non-invasive biomarker classes that may reflect inflammatory activity and disease phenotypes in conditions like endometriosis or adenomyosis [22,85,86].

The feasibility of home-based sampling using menstrual cups supports the potential for large-scale implementation [70,74]. Unlike biopsy-based methods, this approach allows for repeated longitudinal monitoring, which could be valuable for assessing treatment response or disease recurrence. For instance, persistent progesterone resistance or inflammatory activity in ME after hormonal therapy could guide personalized adjustments in management.

### 4.3. Economic Aspects

The potential cost advantage of ME testing is driven less by the assay price alone (in Germany, a single serum biomarker ELISA- or PCR-based test costs approximately 15–25 EUR) and should not be directly compared with the costs of complex endometriosis surgery. The main economic benefit is more likely to come from reducing avoidable invasive procedures and from shortening diagnostic delay and enabling earlier therapy initiation. An earlier non-invasive diagnosis would reduce not only patients’ symptom burden but also their considerable personal expenses, which, in Germany, average 4234 EUR per year, comprising direct costs (e.g., outpatient care, pain therapy, fertility treatments, hormone therapies) estimated at 2060 EUR and indirect costs (e.g., income loss or costs related to comorbidities) estimated at 2174 EUR annually [87].

### 4.4. Strengths and Limitations

To our knowledge, this is the most comprehensive synthesis of studies specifically evaluating ME as a key specimen in endometriosis research. By integrating diagnostic outcomes with mechanistic evidence, we demonstrate that high-performing assays (e.g., aromatase, IGFBP1) correspond directly to the underlying pathology of progesterone resistance and impaired decidualization.

The present systematic synthesis supports the “seed and soil” paradigm of endometriosis pathogenesis, in which Sampson’s concept of retrograde menstruation and Leyendecker’s TIAR hypothesis represent sequential stages rather than competing models. ME differs fundamentally from peripheral blood [67,77], as it constitutes a proinflammatory, proangiogenic, and clonogenic “charged” biofluid, further capable of modifying the peritoneal “soil” through immune modulation and extracellular signaling [49,52].

The limitations of included studies include modest sample sizes and no prospective or longitudinal data. The reported research span over three decades (1990–2025), during which analytical technologies evolved substantially from radioimmunoassay-based protein quantification [81,82] to single-cell transcriptomics [49,58,61] and digital droplet ELISA platforms [50]. This technological heterogeneity limits direct comparability of diagnostic metrics but demonstrates consistent biological signals—particularly elevated inflammatory, adhesive, and hormonal markers—across diverse analytical platforms.

### 4.5. Future Directions

Clinical translation is currently limited by methodological heterogeneity and the lack of comparison with current first-line modalities like TVS or MRI. Integration into clinical guidelines will require methodological standardization, including protocols for sample collection, pre-analytical processing, and harmonization of analytical assays (ELISA, RT-qPCR, and ICC). Standardization should address pre-analytical sample stability resulting from collection technique. Across studies, ME was obtained via menstrual cups, pads, aspiration, or catheter-based sampling, yet key parameters (cycle day, dwell time in a collection device, transport temperature, time-to-processing, and anticoagulants/preservatives) were inconsistently reported. Pre-analytical conditions may differentially affect biomarker classes. While RNA-based biomarkers show acceptable stability in peripheral blood/serum [22,86,88], data regarding other types of biomarkers in menstrual blood are conflicting [89,90,91]. For this reason, further research should address the optimal and standardized time and form of self-collection of ME, maintaining the sampling simplicity, but ensuring that the collection technique, transport, and pre-analytical processing would not influence the sample stability and diagnostic capacity.

Second, prospective, multi-center validations in real-world populations are critical for ensuring reliability and reproducibility of ME-based diagnosis. Future studies must move beyond case–control designs to assess diagnostic performance in unselected symptomatic women, adolescents, and those with early-stage disease, ideally in direct comparison with first-line imaging. Third, the dynamic nature of ME offers a unique opportunity for longitudinal monitoring. Studies should evaluate intra-individual stability and biomarker changes in response to hormonal or surgical therapy for tracking treatment success or recurrence. Longitudinal analyses should identify key determinants of within-person biomarker variation (e.g., cycle characteristics, inflammation, medication, and comorbidities). Fourth, diagnostic precision may be enhanced by integrating multi-marker approaches that combine complementary signatures (e.g., immune, lipidomic, and RNA-based markers) [22,85,92]. Finally, mechanistic insights—such as progesterone resistance and immune dysregulation—should be applied to develop predictive biomarkers for personalized management, guiding treatment selection and risk stratification. In parallel, targeting upstream uterine drivers and peritoneal conditioning steps may represent future disease-modifying therapeutic approaches. Evaluating the health–economic impact and patient acceptability will further support the integration of ME testing into routine care.

## 5. Conclusions

This systematic review demonstrates that ME provides a feasible, patient-acceptable, and pathophysiologically relevant source of diagnostic and mechanistic information in endometriosis. Across 35 studies, ME analysis reveals signatures mirroring intrinsic eutopic endometrial abnormalities, immune dysregulation, angiogenic alterations, progesterone resistance, impaired decidualization, and cellular senescence. These findings confirm that ME reflects core disease biology rather than merely representing shed menstrual debris.

Diagnostically, several biomarkers related to hormonal regulation and stromal-cell function (such as aromatase mRNA, TGF-β1, and IGFBP1) demonstrated very good discriminatory accuracy, with AUC values exceeding 0.90 in controlled settings, while additional modalities—including a lipidomic two-marker model—showed promising accuracy in the moderate-to-high range. Clinical translation now requires prospective validation in real-world populations and direct comparison with established imaging modalities. With methodological optimization, ME-based testing can offer a pathway to shift the diagnostic paradigm from late-stage surgical confirmation toward early, non-invasive, and personalized detection strategies.

## Figures and Tables

**Table 1:** General Characteristics of Included Studies.

First Author (Year)	Study Design	Cases/Controls	Confirmation	ME Collection & Timing	Analytical Methods	Main Focus	Summary of Key Findings
Cadle, et al. (2025) [48]	Case–control	3/4	LSC	Day 2; menstrual cup	Stromal cell isolation; γH2AX; alkaline comet assay; WB	Mechanistic	Impaired DNA damage response and genomic instability in eutopic endometrial stromal cells
Wilson, et al. (2025a) [49]	Case–control	14/19	LSC	Day 1 and 2; menstrual cup	Spectral flow cytometry, cytology, proteomics	Mechanistic	↑ aged neutrophils, ↑ anti-inflammatory macrophages, impaired clearance pathways
Delenko, et al. (2025) [50]	Case–control	Total *n* = 8 *	LSC	Not reported; menstrual cup	Stromal culture, senescence markers (NanoJagg), WB	Mechanistic	↑ cellular senescence in endometriosis eSCs; senolytics (e.g., quercetin) restore function
Wang, et al. (2025) [51]	Case–control	20/20	LSC	Day 2 (2 h collection); menstrual cup (2 mL)	ddELISA, scRNA-seq	Diagnostic (exploratory)	↑ OPN, IL-10, IL-6 in ME; ddELISA platform developed
Gurung, et al. (2025) [52]	Case–control	8/9	Self-reported LSC (cases)	Day 2 (4–6 h); menstrual cup	sEV isolation, TMT proteomics, functional assays	Mechanistic	Altered sEV proteome; ↑ immune activation (↑ CD86) in ME-sEVs; decrease in cellular resistance and junctional protein expression
Wilson, et al. (2025b) [53]	Case–control + mouse model **	10/13	LSC	Day 1 (6–10 h); menstrual cup	Neutrophil immunophenotyping (flow cytometry)	Mechanistic	↑ aged & proangiogenic neutrophils; NETs promote early lesion adhesion
Delenko, et al. (2024) [54]	Case–control	*n* = 3–8 per assay	LSC	Day 1; Not reported	Phosphokinase arrays; WB; flow cytometry; ELISA; scRNA-seq	Mechanistic	Quercetin enhances decidualization in control/endometriosis eSCs via AKT/ERK suppression, p53 activation, senescent-cell apoptosis
Amanda, et al. (2024) [55]	Case–control	20/20	LSC + US	Day 2–3; pad → dried blood spots	RT-qPCR	Diagnostic	Aromatase AUC 0.977; SF-1 AUC 0.862, HSD17B2 AUC 0.807
Starodubtseva, et al. (2024) [56]	Case–control	23/16	LSC	Day 2–3; Cusco speculum → dried blood spots	Lipidomics (HPLC-MS)	Diagnostic	2-lipid panel (PE P-16:0/18:1 + CL 16:0_18:0_22:5_22:6): sens 81%, spec 85%
Wang, et al. (2024) [57]	Case–control	20/10	LSC	Day 2; menstrual cup	Functional assays, WB	Mechanistic	↑ OPN in EM eSCs; OPN knockdown inhibits necroptosis and inflammatory factor release via RhoA-ROS (therapeutic potential)
Febriyeni, et al. (2024) [58]	Case–control	18/17	LSC	Day 2–3; pad → dried blood	RT-qPCR + pyrosequencing (methylation)	Diagnostic	↑ CXCL16 mRNA expression (2.42 times); ↓ CXCL16 DNA methylation in endometriosis-ME
Schwalie, et al. (2024) [59]	Case–control	7/11	LSC	Day 2; menstrual cup	scRNA-seq (CD45^+^/CD45^−^ sorted)	Mechanistic (proof of principle)	↓ decidualisation, ↓ apoptosis, ↑ proliferation, altered immune-stromal crosstalk
Effendi, et al. (2023) [60]	Case–control	40/10	LSC	Day 1–3; menstrual cup	ELISA (TGF-β1)	Diagnostic	AUC = 0.973 at 515 ng/mL (cut-off); sens 80%, spec 90% ^§§^
Ji, et al. (2023) [61]	Case–control	8/8	LSC	Day 1–3; syringe (2 mL ME from Cx)	DIA-proteomics, ELISA validation	Diagnostic (exploratory); mechanistic	↑ CXCL5; ↑ IL1RN in endometriosis; no metrics of diagnostic accuracy
Davoodi Asl, et al. (2023) [62]	Case–control + Experimental-therapeutic	5/10	LSC	Day 2–3; Pipelle catheter	Exosome isolation; RT-qPCR; ELISA; ICC; Annexin V/PI; scratch assay	Mechanistic (therapeutic)	NE-MenSC-derived exosomes ↓ inflammation, ↓ proliferation, ↓ migration, ↓ angiogenesis, ↓ β-catenin, ↑ stemness markers, ↑ apoptosis
Shih, et al. (2022) [63]	Case–control	11/9 ***	LSC	Day 1–2 (4–8 h); menstrual cup/sponge (2.5–10 mL)	scRNA-seq (CD45^−^ stromal)	Mechanistic	↓ NK cells; ↑ pro-inflammatory, ↑ senescent phenotypes, ↓ IGFBP1, ↓ decidualization of eSCs in endometriosis
Miller, et al. (2022) [64]	Case–control	14/19	LSC	Day 1–2; menstrual cup	Multiparameter flow cytometry; transcriptomic analysis	Mechanistic	↓ Th17 cells, ↓ macrophage, ↓ TGF α in ME (endometriosis); dysregulated expression of 47 genes of the Th17 axis and macrophage signaling/activation axis
Sahraei, et al. (2022) [65]	Case–control	3/3	LSC	Day 2–3; sampling catheter (2 mL from Cx)	Flow cytometry + RT-qPCR	Mechanistic	↑ CD10, ↓ CD9; ↑ Cyclin D1, MMP-2, MMP-9, VEGF, IL-1β, IL-6, IL-8, NF-*κ*B; ↓ β-catenin ^§^
Schmitz, et al. (2021) [66]	Case–control	12/11	LSC	Day 1–2 (2 × 12 h); menstrual cup	Flow cytometry (perforin^+^ CD8^+^ T cells)	Mechanistic	↓ cytotoxic potential of T-cell function in ME → reduced elimination of endometriotic cells at ectopic locations
Masuda, et al. (2021) [67]	Case–control	32/29	LSC	Day 2–3; syringe (5 mL ME from Cx)	In vitro assay, ICC, flow cytometry	Mechanistic	Retrograde shedding of clonogenic endometrial cells, SUSD2+ mesenchymal stem cells and N-cadherin+ epithelial progenitor cells into the pelvic cavity as the initial step of endometriosis
Anwar, et al. (2021) [68]	Case–control	30/30	LSC	Day 3; syringe (ME from the posterior fornix)	ICC (VEGF H-score)	Diagnostic	Supports the role of VEGF in endometriosis; but low diagnostic accuracy: AUC 0.672, sens 40%, spec 93.3%
Manan, et al. (2021) [69]	Case–control	38/7	Self-reported/(symptom profile)	Not reported	ELISA (VEGF-A)	Diagnostic	Supports the role of VEGF in endometriosis; Good diagnostic accuracy of VEGF-A: AUC 0.853, sens 84.2%, spec 85.7%.
Nayyar, et al. (2020) [70]	Case–control	24/23 ****	LSC	Day 1–2; menstrual cup/sponge	Functional decidualization assay (IGFBP1 ELISA)	Diagnostic	↓ IGFBP1/↓ decidualization capacity of ME-derived stromal fibroblast cells; Accuracy: AUC 0.92; sens 87.5%, spec 91.7%
Madjid, et al. (2020) [71]	Case–control	30/38	LSC	Day 2–3; 20 drops; collection method not reported	ICC (MMP-9/TIMP-1)	Mechanistic	↑ MMP-9 in endometriosis; TIMP-1 expression inversely related to endometriosis
Mangalonggak, et al. (2020) [72]	Case–control	27/25	LSC or open surgery	Day 1 or 3; menstrual cup	ELISA (Endoglin)	Diagnostic (explorative)	↑ Endoglin in endometriosis; diagnostic potential; role of angiogenesis in EM
Madjid, et al. (2019) [73]	Case–control	63/86	LSC	Day 1–3; 20 drops; collection method not reported	ICC (MMP-9, caspase-3, caspase-9)	Diagnostic (explorative)	No significant differences for caspase-3, caspase-9 and MMP-9 between cases and controls, but increased caspase-3/caspase-9 ratio in endometriosis.
Warren, et al. (2018) [74]	Case–control	7/7	Self-reported LSC (cases)	Day 1–3; menstrual cup	Flow cytometry; scRNA-seq; decidualization assay (IGFBP1)	Mechanistic	↓ uterine uNK cells; ↓ decidualization of stromal fibroblasts in endometriosis
Anwar, et al. (2018) [75]	Case–control	21/21	LSC	Day 1–2; not reported	RT-qPCR (PR-B mRNA)	Diagnostic	↓ PR-B in ME of cases vs. controls; ↓ PR-B in advanced vs. mild endometriosis; Diagnostic utility: sens 90.5%, spec 81.0%
Madjid, et al. (2015) [76]	Case–control	34/48	LSC or open surgery	Day 1–2; not reported	ICC (caspase-3/9, MMP-9)	Mechanistic	↓ caspase-3/9 (reduced apoptosis), trend to ↑ MMP-9
da Silva, et al. (2014) [77]	Case–control	10/7	LSC	Day 1–4; syringe (aspiration from Cx)	ELISA (TNF-α, VEGF), enzymatic methods (NAG, MPO)	Mechanistic	↑ local NAG and MPO activity in cases (ME > peripheral blood), but not in controls; no differences for TNF-α, VEGF, NAG, and MPO between cases and controls
Nikoo, et al. (2014) [78]	Case–control	6/6	LSC	Day 2; menstrual cup	Flow cytometry; RT-qPCR; proliferation, invasion, adhesion assays; WB; ELISA (cytokines)	Mechanistic	E-MenSCs morphologically different (circular, formed 3D aggregates). ↑ CD9, CD10, CD29 expression. ↑ proliferation, ↑ invasion. ↑ IDO1/COX-2; ↓ FOXP3. ↑ IFN-γ, ↑ IL-10, ↑ MCP-1 in co-cultures.
Griffith, et al. (2010) [79]	Case–control (in vitro)	21/8	LSC or open surgery	Day 1–2; Pipelle aspiration	Adherence assay; dot-blot (CD44 splice variants)	Mechanistic	ESCs from endometriosis show significantly ↑ adherence to PMCs (43% vs. 32%, *p* < 0.002). EECs show ↑ adherence (23% vs. 15%, *p* = 0.07). Endometriosis cells more likely to express CD44v6, v7, v8, v9.
Malik, et al. (2006) [80]	Case–control	16/16	LSC	Day 2; menstrual cup	ELISA (VEGF-A, MMP2/9, sFlt)	Mechanistic	No differences between cases and controls; ↑ VEGF-A and MMP in the peritoneal fluid and endometriotic lesions interpreted as “secondary event” unrelated to the endometrium (contradictory to later studies).
Abu-Musa, et al. (1992) [81]	Case–control	28/27	LSC or open surgery	Day 3; syringe (1 mL vaginal aspiration)	RIA	Diagnostic	CA-125 ≥ 72,000 U/mL: sens 89.3%, spec 96.3% for endometriosis in chronic pelvic pain patients. Stage-specific sens: Stage I 85.7%, Stage II 85.7%, Stages III/IV 92.8%.
Takahashi, et al. (1990) [82]	Case–control	38/66	LSC or open surgery	Day 3; syringe (1 mL vaginal aspiration)	RIA	Diagnostic	CA-125 in ME significantly ↑ in all endometriosis stages vs. controls. CA-125 > 100,000 U/mL: sens 65.7%, spec 89.3%.

LSC—laparoscopy; US—ultrasound; Cx—cervix; ICC—immunocytochemistry; RIA—radioimmunoassay; WB—Western blotting; RT-qPCR—Reverse Transcription Quantitative Polymerase Chain Reaction; scRNA-seq—single-cell RNA sequencing; ELISA—enzyme-linked immunosorbent assay; ddELISA—digital droplet ELISA; ↑—increase, upregulation; ↓—decrease, downregulation; MMP-9—Matrix Metalloproteinase-9; TIMP-1—Tissue Inhibitor of Metalloproteinases-1; γH2AX—Phosphorylated histone H2AX (gamma-H2AX); IGFBP1—Insulin-like Growth Factor Binding Protein 1; VEGF-A—Vascular Endothelial Growth Factor A; MPO—myeloperoxidase; NAG—N-acetyl-b-D-glucosaminidase. * The Methods section mentions participants with and without endometriosis within the study group consisting of eight participants, but does not inform about the proportion of cases:controls; ** Human ME used for neutrophil phenotyping; mouse model used only for mechanistic validation; *** the numbers of participants vary depending on the test; **** the numbers of participants vary depending on the test; ^§^ Ref. [65] contains internal discrepancy: abstract states decreased β-catenin, while the Results and Figure 9 report a significant (19-fold) *up*regulation. ^§§^ Effendi et al. [60] report 80% sensitivity and 90% specificity for TGF-β1 at 515 ng/mL in the text and 2 × 2 table, but Table 4 erroneously inverts these values (90%/80%).

**Table 2:** Diagnostic Accuracy of ME-Based Tests.

First Author (Year)	Biomarker(s)	Analytical Platform	Cut-Off	AUC (95% CI)	Sensitivity (%)	Specificity (%)	Sample Size (Cases/Controls)
Amanda, et al. (2024) [55]	Aromatase	RT-qPCR	>1.63 (ΔΔCt)	0.977 (0.929–1.000) *	95	90	20/20
Amanda, et al. (2024) [55]	SF-1	RT-qPCR	>1.71 (ΔΔCt)	0.862 (0.744–0.980) *	90	80	20/20
Amanda, et al. (2024) [55]	HSD17B2	RT-qPCR	>1.83 (ΔΔCt)	0.807 (0.670–0.944) *	80	75	20/20
Starodubtseva, et al. (2024) [56]	Two-lipid predictive model (CL 16:0_18:0_22:5_22:6 + PE P-16:0/18:1)	HPLC-MS lipidomics	Probability > 0.59	0.870 (0.759–0.981) *	81	85	23/16
Effendi, et al. (2023) [60]	TGF-β1	ELISA	≥515 ng/mL	0.973 (0.928–1.000)	80	90 ^§^	40/10
Anwar, et al. (2021) [68]	VEGF	ICC (VEGF H-score)	H-score threshold > 6	0.672 (0.535–0.809) *	40	93.3	30/30
Manan, et al. (2021) [69]	VEGF-A (menstrual blood)	ELISA	347 pg/mL	0.853 (0.716–0.941)	84.2	85.7	38/7
Nayyar, et al. (2020) [70]	IGFBP1 (functional decidualization assay)	ELISA (functional assay on ME-SFCs)	Not reported	0.920 (0.838–1.000) *	87.5	91.7	24/23
Anwar, et al. (2018) [75]	PR-B mRNA (relative expression)	RT-qPCR	≤1.1355 (μg/dL) ^†^	Not reported	90.5	81.0	21/21
Abu-Musa, et al. (1992) [81]	CA-125	RIA	≥72,000 U/mL	Not reported	89.3	96.3	28/27
Takahashi, et al. (1990) [82]	CA-125	RIA	>100,000 U/mL	Not reported	65.7	89.3	38/66 ^§§^

RT-qPCR—reverse transcription quantitative polymerase chain reaction; ELISA—enzyme-linked immunosorbent assay; ICC—immunocytochemistry SF-1—Steroidogenic Factor-1 (NR5A1); HSD17B2—Hydroxysteroid 17-Beta Dehydrogenase Type 2; IGFBP1—Insulin-like Growth Factor Binding Protein 1; PR-B—Progesterone Receptor Isoform B; VEGF-A—Vascular Endothelial Growth Factor A; CL—Cardiolipin; PE P-16:0/18:1—Plasmenylphosphatidylethanolamine; and RIA—radioimmunoassay. ^§^ Effendi et al. [60] report 80% sensitivity and 90% specificity for TGF-β1 at 515 ng/mL in the text and 2 × 2 table, but Table 4 erroneously inverts these values (90%/80%); ^§§^ 38 endometriosis patients were tested against 30 healthy controls and 36 patients had non-endometriotic pelvic pathology. ^†^ Anwar et al. [75] report RT-qPCR-derived PR-B mRNA expression in μg/dL, an unusual unit for gene expression data. Internal labeling inconsistencies (axis label: “progesterone concentration”; text: “progesterone levels”) suggest possible conflation of PR-B receptor expression with progesterone hormone levels. * 95% CIs estimated from AUC and sample sizes using the Hanley–McNeil method.

**Table 3:** Pathophysiological Alterations in ME of Women with Endometriosis.

First Author (Year)	Pathobiological Mechanism	Key Mechanistic Findings	Implications for Pathogenesis
Cadle, et al. (2025) [48]	DNA damage & genomic instability	↑ γH2AX foci; impaired comet assay repair; altered ATM/ATR/BRCA1 signaling	Genomic instability in eutopic endometrium may facilitate establishment of endometriotic lesions
Wilson, et al. (2025a) [49]	Innate/adaptive immune dysregulation	↑ aged neutrophils (CXCR4+), ↑ anti-inflammatory macrophages, ↑ T helper cells; ↓ cytotoxic T cells; ↓ proinflammatory antigen-presenting macrophages; altered proteomic pathways	Immune imbalance with premature neutrophil aging and macrophage polarization may alter the microenvironment of the peritoneal cavity promoting endometriosis development
Delenko, et al. (2025) [50]	Cellular senescence	↑ SA-β-gal, p16, p21; impaired p53 response; rescued by quercetin/senolytics	Premature senescence contributes to stromal dysfunction and impaired decidualization
Wang, et al. (2025) [51]	Multi-protein inflammatory signature	ddELISA: ↑ OPN, IL-10, IL-6 in endometriosis	Reflects systemic and local immune dysregulation; supports ME as surrogate for endometrial status
Gurung, et al. (2025) [52]	EV-mediated signaling	5000+ proteins identified; ↓ immune/repair proteins (e.g., complement, integrins); ↑ IgM, CD86	EVs may propagate pro-inflammatory and barrier-disruptive signals to peritoneum
Wilson, et al. (2025b) [53]	Innate immune activation	↑ aged (CD16−CXCR2+) & pro-angiogenic (VEGFR1+) neutrophils; ↑ NET markers (MPO, ELA2); ↑ fibrinogen-mediated adhesion	Neutrophils contribute to a permissive proinflammatory peritoneal microenvironment and promote early lesion attachment/adhesion
Delenko, et al. (2024) [54]	Decidualization & stress signaling	Quercetin ↑ decidualization via ↓ AKT/ERK, ↑ p53; selectively targets senescent-like cells	Decidualization defect is partially reversible; links senescence to progesterone resistance
Amanda, et al. (2024) [55]	Steroidogenesis & progesterone resistance	↑ Aromatase, ↑ SF-1; ↑ HSD17B2 in ME	Local dysregulation of estrogen synthesis in endometriosis: ↑expression of aromatase, SF-1, and HSD17B2 in ME, but not in endometrial biopsy—highlights the role of ME, which is non-identical with eutopic endometrium
Starodubtseva, et al. (2024) [56]	Altered plasmalogen and cardiolipin composition	Significant shifts in ether-linked PE species and cardiolipins	Suggests mitochondrial lipid disturbance and altered membrane dynamics
Wang, et al. (2024) [57]	Osteopontin-driven invasion	↑ OPN → activates RhoA/ROS → ↑ migration/invasion; inhibition reduces phenotype	OPN is a key driver of invasive behavior in eutopic endometrium
Febriyeni, et al. (2024) [58]	Chemokine signaling & epigenetics	↑ CXCL16 mRNA; ↓ DNA methylation	Epigenetic dysregulation of CXCL16 (hypomethylation with increased expression) may contribute to inflammatory/chemokine signaling in endometriosis
Schwalie, et al. (2024) [59]	Immune-stromal crosstalk	Altered uNK, macrophage, and stromal states; disrupted ligand-receptor networks	Reflects chronic inflammatory microenvironment in eutopic endometrium
Effendi, et al. (2023) [60]	TGF-β signaling	↑ TGF-β1 (515 ng/mL cut-off)	Higher expression of ↑ TGF-β1 can be associated with altered proliferation, apoptosis, differentiation, and immune response in endometriosis
Ji, et al. (2023) [61]	Inflammatory mediators	↑ CXCL5; ↑ IL1RN	Imbalance in neutrophil recruitment vs. anti-inflammatory regulation; suggests CXCL5 and IL1RN as potential biomarkers
Davoodi Asl, et al. (2023) [62]	Exosome-mediated therapeutic modulation	NE-MenSC exosomes → ↓ E-MenSC expression of: inflammation (IL-6, IL-8, IL-1β, COX-2, NF-κB, HIF1α, TNF-α), proliferation (cyclin D1, Ki67), migration (MMP-2, MMP-9), angiogenesis (VEGF), β-catenin. Induced apoptosis (↑ BAX/BCL-2); ↑ stemness (OCT-4, NANOG, SOX-2).	Proof-of-concept that NE-MenSC-derived exosomes can reverse the pathological phenotype of E-MenSCs; therapeutic potential for ME-derived cell-free approaches
Shih, et al. (2022) [63]	Stromal decidualization defect	↓ NK cells; ↑ pro-inflammatory and senescent phenotypes; ↓ IGFBP1 and LEFTY2, DCN, MDK and other progesterone sensitive gene markers in eSCs in endometriosis	Pro-inflammatory and senescent eSC phenotypes in endometriosis; conversely, decidualized eSCs show abundant IGFBP1 mRNA plus LEFTY2, DCN, LUM, MDK, C1QTNF6, APOE/D and other progesterone-sensitive decidualization/fertility markers.
Miller, et al. (2022) [64]	Th17-macrophage axis	↓ Th17 cells; ↓ tissue-resident macrophages; altered polarization	Adaptive-innate immune imbalance promotes chronic inflammation
Sahraei, et al. (2022) [65]	Inflammatory gene expression	↑ IL-1β, IL-6, IL-8, NF-κB, SOX-2, MMP-2, MMP-9, VEGF; ↓ β-catenin; ↓ BAX/BCL-2 ratio (reduced apoptosis)	Differential expression of genes associated with inflammation, apoptosis, migration, and angiogenesis in eSCs prior to retrograde menstruation
Schmitz, et al. (2021) [66]	Cytotoxic T-cell dysfunction	↓ perforin^+^ CD8^+^ T cells in endometriosis	↓ cytotoxic potential of T-cell function in ME → impaired local immune surveillance at ectopic locations
Masuda, et al. (2021) [67]	Stem/progenitor cells	Clonogenic endometrial cells represented with ↑ frequency (eMSC 76.9% vs. 44.4%; eEPC 60.0% vs. 25.0%) and at ↑ concentrations in peritoneal fluid of women with endometriosis. No clonogenic eSCs in peripheral blood.	Supports the role of shed clonogenic endometrial cells, eMSCs and eEPCs, in the pathogenesis of endometriosis
Anwar, et al. (2021) [68]	Angiogenesis (VEGF)	↑ VEGF H-score in endometriosis	Supports angiogenic priming of shed endometrium
Manan, et al. (2021) [69]	Angiogenesis (VEGF-A)	↑ VEGF-A	Confirms pro-angiogenic state in ME
Nayyar, et al. (2020) [70]	Decidualization defect	↓ IGFBP1 and ↓ ALDH1A1 gene expression, ↑ podoplanin surface expression; shift of normal eSCs to endometriosis-like phenotype when stimulated with TNF and IL-1b	Endometriosis-like ME phenotype is characterized by ↓ decidualization capacity, ↑ cell migration and can be reproduced in normal stromal cells by exposure to inflammatory cytokines
Madjid, et al. (2020) [71]	ECM remodeling	↑ MMP-9, ↓ TIMP-1 in endometriosis	Matrix remodeling as a part of endometriosis pathogenesis; vague predictive potential of MMP-9/TIMP-1 ratio
Mangalonggak, et al. (2020) [72]	Angiogenesis (Endoglin)	↑ Endoglin in endometriosis	Adds to evidence of vascular activation in endometriosis
Madjid, et al. (2019) [73]	Cytomorphology	Altered cell morphology and marker expression	Supports cellular-level abnormalities detectable via simple cytology
Warren, et al. (2018) [74]	Decidualization defect	↓ uterine uNK cells; ↓ decidualization potential of stromal fibroblasts; ↓ IGFBP-1 production following cAMP- and vehicle-treatment in endometriosis	Impaired decidualization of eSCs in endometriosis
Anwar, et al. (2018) [75]	Progesterone resistance (PR-B)	↓ PR-B expression in cases vs. controls; ↓ PR-B expression in advanced vs. mild endometriosis	Molecular correlate of impaired progesterone signaling
Madjid, et al. (2015) [76]	Apoptosis & ECM	↓ Caspase-3, Caspase-9, ↑ MMP-9	Combined apoptosis and matrix dysregulation in shed tissue
da Silva, et al. (2014) [77]	Inflammation & angiogenesis	↑ IL-6, TNF-α, VEGF, MCP-1	Confirms pro-inflammatory, pro-angiogenic ME milieu
Nikoo, et al. (2014) [78]	Stem cell phenotype, invasion, immunomodulation	E-MenSCs: ↑ CD9, CD10, CD29; more circular morphology; formed 3D aggregates; ↑ proliferation; ↑ invasion; ↑ IDO1/COX-2 gene & protein; ↓ FOXP3; ↑ IFN-γ, IL-10, MCP-1 in co-cultures	Inherent phenotypic and functional differences in E-MenSCs support both the retrograde menstruation and stem cell theories; E-MenSCs exhibit a biological program combining invasiveness, immune evasion, and inflammatory activation
Griffith, et al. (2010) [79]	Cellular adhesion & CD44 splice variants	↑ ESC adherence to PMCs (43% vs. 32%, *p* < 0.002); ↑ EEC adherence (23% vs. 15%, *p* = 0.07); ↑ expression of CD44v6, v7, v8, v9 in endometriosis endometrial cells	Increased eutopic endometrial-peritoneal adherence may contribute to the formation of endometriotic lesions; CD44 splice variant expression may facilitate initial attachment to mesothelium via hyaluronan binding
Malik, et al. (2006) [80]	Angiogenesis & proteolysis	No significant differences in VEGF-A, MMP-2, MMP-9, sFlt	Highlights heterogeneity/contradictory to later studies; not all studies replicate angiogenic findings.

ICC—immunocytochemistry; ELISA—enzyme-linked immunosorbent assay; EVs—extracellular vesicles; uNK—uterine natural killer cells; E-MenSCs—menstrual stromal stem cells in endometriosis; NE-MenSCs—non-endometriosis menstrual stromal stem cells eMSCs—endometrial mesenchymal stromal cells; eEPCs—endometrial epithelial progenitor cells; OPN—osteopontin; SSEA-1—stage-specific embryonic antigen-1 (stem/progenitor-cell marker); γH2AX—phosphorylated histone H2AX, marker of DNA double-strand breaks; HMGB1—high-mobility group box 1; LOD—limit of detection; ATM/ATR—ataxia telangiectasia mutated/ATM- and Rad3-related; BRCA1—breast cancer gene 1; HMGB1—high-mobility group box 1 protein; SA-β-gal—senescence-associated β-galactosidase; SF-1—steroidogenic factor-1 (NR5A1); TNF-α—tumor necrosis factor-α; TGF-β1—transforming growth factor beta 1; HSD17B2—17β-hydroxysteroid dehydrogenase type 2; EpCAM—epithelial cell adhesion molecule; IDO1—indoleamine 2,3-dioxygenase-1; COX-2—cyclooxygenase-2; FOXP3—forkhead transcription factor-3; IFN-γ—interferon-γ; EECs—endometrial epithelial cells; ESCs—endometrial stromal cells; PMCs—peritoneal mesothelial cells (PMCs); VEGF-A—vascular endothelial growth factor A; PR-B—progesterone receptor B; MMP-2—matrix metalloproteinase-2; MMP-9—matrix metalloproteinase-9; and sFlt—soluble fms-like tyrosine kinase-1.

## Data Availability

No new data were created or analyzed in this study.

## Supplementary Materials

The following supporting information can be downloaded at: https://www.mdpi.com/article/10.3390/diagnostics16050677/s1, PRISMA 2020 Checklist.

## Appendix A

**Table A1:** Risk of bias and quality assessment of included studies.

First Author (Year)	Study Type	Risk-of-Bias Tool	Overall Risk of Bias	Key Methodological Limitations
Cadle, et al. (2025) [48]	Mechanistic case–control (ME-derived stromal cells)	NIH	Moderate	Small sample (3/4); convenience sampling; limited reporting on blinding; lab endpoints only
Wilson, et al. (2025a) [49]	Mechanistic (flow cytometry, proteomics)	NIH	Moderate	Moderate cohort (14/19); possible selection bias; confounders (age, race) not fully adjusted
Delenko, et al. (2025) [50]	Mechanistic senescence (ME stromal cells)	NIH	Moderate	Sample size not explicitly stated (total *n* of participants = 8); no blinding reported; external validity limited
Wang, et al. (2025) [51]	Exploratory multiplex biomarker panel	NIH	Moderate–High	Cross-sectional; small sample (20/20); exploratory biomarker discovery; no diagnostic validation or threshold definition planned
Gurung, et al. (2025) [52]	Mechanistic EV study (ME sEVs)	NIH	Moderate	Small sample (8/9); symptomatic controls; limited clinical characterization
Wilson, et al. (2025b) [53]	Immune mechanistic (neutrophils in ME)	NIH	Moderate	Case–control (10/13); cross-sectional; no functional validation in humans
Delenko, et al. (2024) [54]	In vitro intervention (quercetin; ME stromal cells)	NIH	Moderate	In vitro model; *n* = 3–8 per assay; no clinical outcomes
Amanda, et al. (2024) [55]	Diagnostic molecular (RT-qPCR)	QUADAS-2	High	Case–control (20/20); no prospective validation; threshold derived internally; unclear blinding
Starodubtseva, et al. (2024) [56]	Diagnostic lipidomics (dried spots)	QUADAS-2	High	Case–control (23/16); AUC 0.87 reported; internal model without external validation; risk of overfitting
Wang, et al. (2024) [57]	Mechanistic OPN study (ME stromal cells)	NIH	Moderate	Case–control (20/10); functional assays only; no diagnostic validation
Febriyeni, et al. (2024) [58]	Molecular biomarker (CXCL16 mRNA/promoter methylation)	NIH	Moderate-High	Case–control (18/17); no ROC; internal threshold
Schwalie, et al. (2024) [59]	scRNA-seq mechanistic (ME)	NIH	Moderate	Small cohort (7/11); no replication
Effendi, et al. (2023) [60]	Diagnostic (TGF-β1 ELISA)	QUADAS-2	Moderate	Case–control design with a small and imbalanced control group (40/10).
Ji, et al. (2023) [61]	Proteomics exploratory (ME)	NIH	Moderate-High	Discovery cohort (8/8); no validation cohort; numerical AUC/sensitivity/specificity not reported
Davoodi Asl, et al. (2023) [62]	Experimental (exosome treatment of E-MenSCs)	NIH	Moderate-High	Small endometriosis group (*n* = 5); in vitro model only (no in vivo/clinical validation)
Shih, et al. (2022) [63]	scRNA-seq (menstrual endometrial tissue)	NIH	Moderate	Very small (11/9); selection bias likely
Miller, et al. (2022) [64]	Immune mechanistic (Th17/macrophages)	NIH	Moderate	Small sample (14/19); single-center
Sahraei, et al. (2022) [65]	Gene expression (ME stromal cells)	NIH	Moderate-High	Very small *n* (3/3); no correction for multiple comparisons
Schmitz, et al. (2021) [66]	Immune cytotoxicity (CD8^+^ perforin^+^)	NIH	Moderate	Case–control (12/11); cross-sectional
Masuda, et al. (2021) [67]	Stem/progenitor-cell study (ME + PF)	NIH	Moderate	Case–control (32/29); mechanistic ME+PF stem/progenitor-cell assays; not designed as diagnostic accuracy study
Anwar, et al. (2021) [68]	Diagnostic ICC (VEGF H-score)	QUADAS-2	High	Case–control (30/30); imbalanced groups; low sensitivity (40%); internal threshold
Manan, et al. (2021) [69]	Diagnostic ELISA (VEGF-A)	QUADAS-2	High	Case–control (38/7); AUC based on 38/7 for ROC → very small control group; histologic confirmation missing (endometriosis only based on clinical symptoms)
Nayyar, et al. (2020) [70]	Diagnostic functional assay (IGFBP1)	QUADAS-2	High	Case–control (24/23); no external validation; internal threshold
Madjid, et al. (2020) [71]	ICC (MMP-9/TIMP-1)	NIH	Moderate-High	Case–control (30/38); semi-quantitative; no ROC
Mangalonggak, et al. (2020) [72]	ELISA (Endoglin/CD105)	NIH	Moderate-High	Case–control (27/25); significant group differences in endoglin levels; no ROC analysis or diagnostic thresholds reported
Madjid, et al. (2019) [73]	ICC (cytology/morphology)	NIH	Moderate-High	Case–control (63/86); exploratory; no accuracy metrics
Warren, et al. (2018) [74]	Functional decidualization assay	NIH	High	Very small (7/7); no blinding; no diagnostic framework
Anwar, et al. (2018) [75]	Diagnostic qRT-PCR (PR-B)	QUADAS-2	High	Case–control (21/21); internal threshold; no validation. Outcome reporting inconsistent (PR-B qRT-PCR described, but results reported in µg/dL and figures refer to progesterone): limited interpretability.
Madjid, et al. (2015) [76]	ICC (caspases, MMPs)	NIH	Moderate-High	Case–control (34/48); semi-quantitative; no correction for multiplicity
da Silva, et al. (2014) [77]	Cytokines/angiogenesis markers	NIH	Moderate	Case–control (10/7); early study; limited confounder control
Nikoo, et al. (2014) [78]	Mechanistic (MenSC phenotyping)	NIH	Moderate	Small sample (6/6); multiple validated methods (flow cytometry, qRT-PCR, functional assays); comprehensive reporting; no blinding
Griffith, et al. (2010) [79]	Mechanistic (in vitro adherence/CD44)	NIH	Moderate	Case–control (21/8); small control group; in vitro model; CD44 variant analysis on subset only (9 vs. 5 for ESCs); no clinical validation
Malik, et al. (2006) [80]	Exploratory (biomarker)	NIH	High	Case–control (16/16); pad-extract method; no significant differences; no blinding
Abu-Musa, et al. (1992) [81]	Diagnostic (CA-125)	QUADAS-2	High	Case–control (28/27); highly selected chronic pelvic pain population; no blinding; no ROC; outdated methodology
Takahashi, et al. (1990) [82]	Diagnostic (CA-125)	QUADAS-2	High	Case–control (38/66; controls: 30 healthy + 36 other pelvic pathology); no blinding; no ROC; threshold (100,000 U/mL) derived post hoc; outdated methodology

NIH—National Institutes of Health (NIH Study Quality Assessment Tool); QUADAS-2—Quality Assessment of Diagnostic Accuracy Studies, Version 2; ICC—Immunocytochemistry; RT-qPCR—Reverse Transcription Quantitative Polymerase Chain Reaction; LFIA—Lateral Flow Immunoassay; VEGF—Vascular Endothelial Growth Factor; MMP-9—Matrix Metalloproteinase-9; TIMP-1—Tissue Inhibitor of Metalloproteinases-1; and PR-B—Progesterone Receptor Isoform B.

**Table A2:** Diagnostic studies without ROC-derived accuracy metrics.

First Author (Year)	Diagnostic Target/Assay	Control Type	Findings with Diagnostic Relevance (No ROC Metrics)	Reason for Exclusion from Table 2
Wang, et al. (2025) [51]	Multiplex ME biomarker panel (OPN, IL-10, IL-6)	Healthy	Several biomarkers differ between groups, but panel was exploratory; no thresholds or accuracy metrics	No ROC, no Sens/Spec, no cut-offs
Wang, et al. (2024) [57]	OPN in ME	Healthy	↑ OPN in cases; functional relevance shown in vitro; no diagnostic modeling	No ROC metrics
Febriyeni, et al. (2024) [58]	CXCL16 mRNA & DNA methylation	Healthy	↑ CXCL16 mRNA expression, DNA hypomethylation; group differences only	No ROC analysis
Schwalie, et al. (2024) [59]	scRNA-seq profiling (immune–stromal states)	Healthy	Altered ME cellular composition; no classifier or diagnostic output	No accuracy metrics
Ji, et al. (2023) [61]	DIA proteomics (CXCL5, IL1RN)	Healthy	Differentially expressed proteins between cases and controls; ROC mentioned but AUC/sensitivity/specificity not reported	No extractable diagnostic metrics
Schmitz, et al. (2021) [66]	Perforin^+^ CD8^+^ T-cell frequency	Healthy	↓ Cytotoxic T cells in endometriosis; biological difference only	No diagnostic performance data
Masuda, et al. (2021) [67]	Stem/progenitor cell clonogenicity (ME + PF)	Healthy	Increased clonogenic potential in ME cells of cases; mechanistic finding, no diagnostic model	Mechanistic; no ROC
Madjid, et al. (2020) [71]	MMP-9/TIMP-1 immunocytochemistry	Healthy	Altered MMP-9/TIMP-1 balance; non-quantitative cytology	No accuracy metrics
Mangalonggak, et al. (2020) [72]	Endoglin (CD105) ELISA	Healthy	↑ Endoglin levels in cases vs. controls; ↑ endoglin levels in severe vs. mild endometriosis, but no ROC or thresholds	No Sens/Spec; no AUC
Madjid, et al. (2019) [73]	Cytomorphology (ICC)	Healthy	No significant differences for caspase-3, caspase-9 and MMP-9 between cases and controls, but increased caspase-3/caspase-9 ratio in endometriosis.	No diagnostic quantification
Warren, et al. (2018) [74]	Decidualization response (IGFBP1)	Healthy	Impaired decidualization in endometriosis; very small *n* (7/7)	No ROC; small sample size
Madjid (2015) [76]	Caspase-3, Caspase-9, MMP-9	Healthy	↓ Caspase-3, caspase-9 and ↑ MMP-9 in cases	No validation as diagnostic assay
da Silva, et al. (2014) [77]	ME enzymes/cytokines (MPO, NGO, TNF-α, VEGF)	Healthy	↑ Local increase in NAG and MPO; no inter-group difference	No ROC analysis
Malik, et al. (2006) [80]	ME VEGF/MMPs; menstrual volume	Healthy	No significant differences between groups	No diagnostic signal

LSC—laparoscopy; ROC—Receiver Operating Characteristic (curve); OPN—Osteopontin; TNF-α—Tumor necrosis factor-α; VEGF—Vascular Endothelial Growth Factor; MMP-9—Matrix Metalloproteinase-9; TIMP-1—Tissue Inhibitor of Metalloproteinases-1; IL1RN—Interleukin-1 Receptor Antagonist; MPO—myeloperoxidase; and NAG—N-acetyl-b-D-glucosaminidase.
